# Review of the Relationship Between the Composition, Strength, and Ultimate Tensile Strain of Engineering Geopolymer Composites

**DOI:** 10.3390/ma18245603

**Published:** 2025-12-13

**Authors:** Xiaomei Wan, Weili Guo, Jiahao Cong, Chen Wang, Mingjin Han

**Affiliations:** 1 School of Civil Engineering, Qingdao University of Technology, Qingdao 266520, China; 15376478015@163.com (W.G.); happywang93@outlook.com (C.W.); 15069753363@163.com (M.H.); 2 Cooperative Innovation Center of Engineering Construction and Safety in Shandong Blue Economic Zone, Qingdao University of Technology, Qingdao 266520, China; 3 Weihai Construction Group Co., Ltd., Weihai 264200, China; x1443302132@163.com

**Keywords:** engineered geopolymer composite, tensile strain, compressive strength, curing regime

## Abstract

Engineered Geopolymer Composites (EGC) combine the high ductility and multi-crack characteristics of traditional Engineered Cementitious Composites (ECC) with the sound low-carbon advantages of geopolymers, making them a research hotspot in the green high-performance materials. This study focuses on the influence of EGC composition (precursor, activator, fiber and fine aggregate) on its tensile properties and the curing regime for different precursor compositions. The reported results data (with ultimate tensile strain exceeding 2%) from recent EGC studies are collected and reviewed. It concludes the systems and mix proportion ranges that are beneficial to tensile properties in current EGC research: blended system of fly ash and slag as the precursor; blended system of sodium hydroxide and water glass (with a modulus ranging from 1.2 to 1.4 and an alkali equivalent from 4% to 8%) as the activator; PE fiber (with a content of 2.0% and an aspect ratio of 500–750) or PVA fiber (with a content of 1.8–2.0% and an aspect ratio of approximately 300) as the reinforced fiber; silica sand (with a particle size of 100–300 μm) as the fine aggregate. Different curing regimes are selected according to different precursor types, and segmented curing and normal-temperature curing are widely adopted currently. This study reveals the relationship between compressive strength and tensile strain. When the EGC matrix strength is in the range of 25–45 MPa, it is easier to achieve excellent ductility. This study provides a theoretical basis and design reference for the material optimization and engineering application of EGC.

## 1. Introduction

In the 1970s, fibers were incorporated into concrete to enhance its tensile performance [1,2,3,4]. In the 1990s, Engineered Cementitious Composites (ECC) were developed [5,6,7]. After the first crack occurs in this material during the tensile process, ECC exhibits strain-hardening behavior. Meanwhile, the cracks formed are characterized by being fine and dense, and ECC possesses a tensile strain capacity hundreds of times higher than that of traditional concrete [8,9,10].

Cement production is an energy-intensive process that contributes approximately 7–8% of global anthropogenic CO_2_ emissions [11,12,13]. Moreover, due to ECC’s incompatibility with coarse aggregates, it typically requires two to three times more cement than ordinary concrete, posing serious environmental concerns and limiting its practical application [14]. Consequently, there is an urgent need to replace or reduce cement content to enhance sustainability. Among various alternatives, geopolymers have emerged as one of the most promising candidates [15,16,17,18]. Geopolymers are inorganic polymers formed via geopolymerization reactions of aluminosili-cate-rich materials—such as fly ash or slag—under alkaline activation. First introduced by Davidovits in the 1970s as a cement substitute [19], geopolymers exhibit similar mechanical properties and superior chemical resistance compared to Portland cement [20,21,22,23].

Professor Victor C. Li, the originator of ECC, defines it as a cementitious composite with ultra-high ductility and tensile strain capacities typically exceeding 2% [8]. Building on this concept, researchers have proposed the development of Engineered Geopolymer Composites (EGC) by integrating ECC principles into geopolymer systems [24,25,26]. Therefore, this review adopts a 2% tensile strain threshold to define effective EGC performance. EGCs retain the strain-hardening and crack-controlling capabilities of ECC while reducing carbon emissions up to 50–80% in best-case scenarios, depending on precursor type and system boundaries [27,28,29], improving chemical durability, and enabling high utilization rates (>60%) of industrial waste [30,31,32,33].

The composition of geopolymers—including raw material types, activator formulations, and tunable parameters such as the Si/Al ratio and modulus—offers considerable design flexibility [24,34,35,36,37]. The introduction of fibers further adds complexity, involving factors such as fiber type, content, and aspect ratio [38,39,40,41,42,43]. As such, a comprehensive understanding and review of current research on EGC formulation and performance is both timely and necessary.

This paper reviews the research progress on the influence of raw materials, activators, fibers, fine aggregates and curing regime on the uniaxial tensile strain behavior of EGC. It presents a statistical analysis of effective data from EGC studies published in the past five years, focusing on systems with an ultimate tensile strain exceeding 2%, a 2% ultimate tensile strain threshold was chosen as the evaluation criterion for this study to quantify whether the tensile performance of geopolymer composites meets the standard(All data are derived from literature published in the past five years in Web of Science and CNKI [China National Knowledge Infrastructure]), with particular attention to the application of different EGC component combinations. Furthermore, it explores the relationship between compressive strength and ultimate tensile strain capacity. Meanwhile, for different precursor combinations, the relationship between matrix strength and ultimate tensile strain capacity of EGC is also investigated (In the pie chart, percentages are rounded to one decimal place, so the total may be slightly off).

## 2. Composition of EGC

### 2.1. Precursor

Geopolymerization refers to the depolymerization–condensation reaction of aluminosilicate precursors—either natural minerals (e.g., metakaolin, zeolite) or industrial by-products (e.g., slag, fly ash)—under alkaline activation, resulting in a three-dimensional inorganic binder. This reaction centers on activating the reactive aluminosilicate components, and different precursors lead to varied reaction kinetics and product structures. For example, low-calcium precursors like fly ash exhibit slow reaction rates and primarily generate N-A-S-H gels (amorphous 3D net-works), while high-calcium precursors like ground granulated blast furnace slag (GGBS) react faster and form layered products such as C-S-H and C-A-S-H gels due to the presence of Ca^2+^ ions [14,44,45,46,47,48,49,50,51,52].

**Figure 1:**
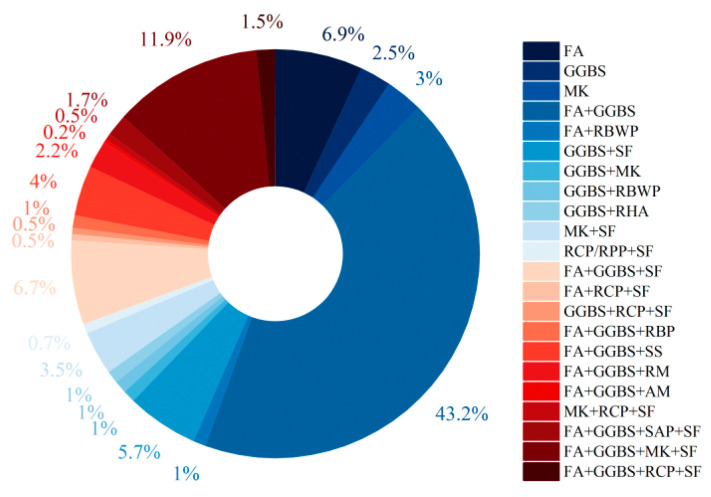
Statistical analysis of the proportion of precursor types in 405 valid EGC research cases reported in the past ten years [37,39,47,51,52,53,54,55,56,57,58,59,60,61,62,63,64,65,66,67,68,69,70,71,72,73,74,75,76,77,78,79,80,81,82,83,84,85,86,87,88,89,90,91,92,93,94,95,96,97,98,99,100,101,102,103,104,105,106,107,108,109,110,111,112,113,114,115,116,117,118,119,120,121]. Note: The order of the legend corresponds to the order of the pie chart sectors (starting clockwise from the 12 o’clock position).

**Figure 2:**
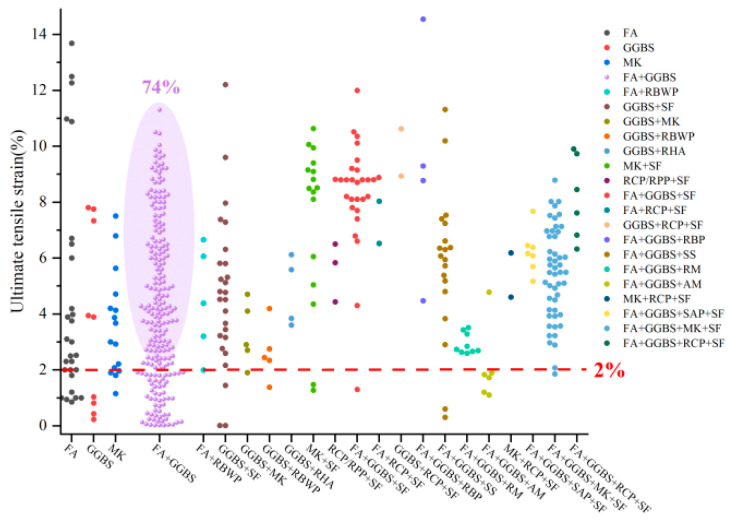
Statistical analysis of the relationship between precursor types and ultimate tensile strain in 474 EGC research cases reported in the past five years [37,39,47,52,53,54,55,56,57,58,59,60,61,62,63,64,65,66,67,68,69,70,71,72,73,74,75,76,77,78,79,80,81,82,83,84,85,86,87,88,89,90,91,92,93,94,95,96,97,98,99,100,101,102,103,104,105,106,107,108,109,110,111,112,113,114].

**Figure 3:**
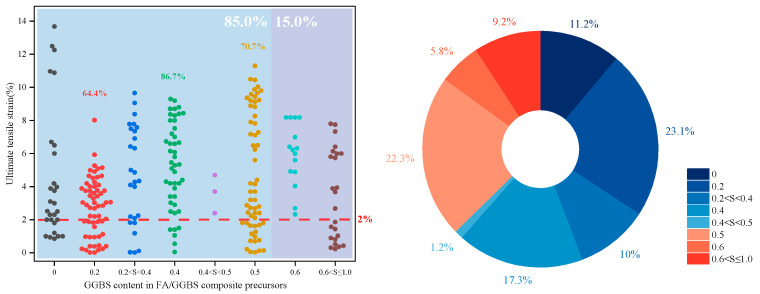
Statistical analysis of the relationship between GGBS content and ultimate tensile strain in 260 FA/GGBS-based EGC research cases reported in the past five years [37,39,47,52,53,54,55,56,57,58,59,60,61,62,63,64,65,66,67,68,69,70,71,72,73,74,75,76,77,78,79,80,81,82,83,84,85,86,87,88,89,90,91,92,93,94,95,96,97,98,99,100,101,102,103,104,105,106,107,108,109,110,111,112,113,114]. Note: In the left figure, the colored percentages indicate the qualification rate with the ultimate tensile strain of 2% as the passing standard. In the right figure, the order of the legend corresponds to the order of the pie chart sectors (starting clockwise from the 12 o’clock position).

As shown in Figure 1, current research trends favor blended precursors combining both low-and high-calcium sources. Among studies from the past five years and eight studies from the last decade reporting EGC with ultimate tensile strains ≥ 2%, only 12.4% employed single precursors (e.g., FA, GGBS, MK), whereas approximately 43.2% used fly ash–slag blends. As shown in Figure 2, these systems demonstrated a tensile performance qualification rate of up to 74% (the past five years). The internal structure of such blends features interwoven N-A-S-H and C-(A)-S-H gels, resulting in a denser and more cohesive matrix compared to systems based on a single gel type [53,54,55]. As shown in Figure 3, in FA-GGBS blended systems, slag content varies from 0% to 100%, with content ≤ 50% accounting for 85% of cases. A slag content of 40% yields the highest qualification rate (86.7%) for tensile strain (set 2% as the passing standard), following a general trend of increasing then decreasing performance with higher slag content.

As shown in Figure 1,recent studies have also explored ternary or quaternary precursor systems, commonly modifying FA-GGBS blends by incorporating silica fume, steel slag or metakaolin. Other researchers have tested red mud, alkali mud (residue from water glass production), and recycled micro-powders. These multi-source blends have also achieved promising tensile strain performance [37,57,58,59,65,66,67,69,70,72,73,74,85,88,90,96,99,100,105,106].

### 2.2. Alkali Activators

**Figure 4:**
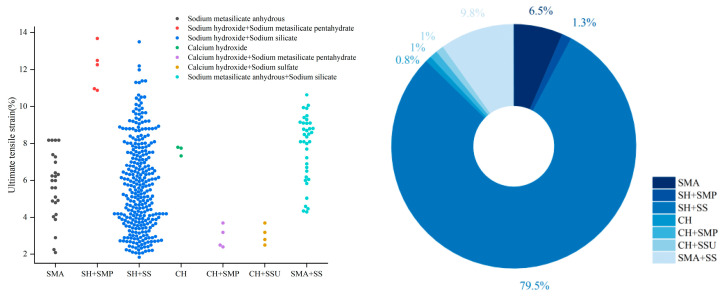
Statistical analysis of the proportion of activator types and the relationship between activator types and ultimate tensile strain in 386 valid EGC research cases reported in the past five years [37,39,47,52,53,54,55,56,57,58,59,60,61,62,63,64,65,66,67,68,69,70,71,72,73,74,75,76,77,78,79,80,81,82,83,84,85,86,87,88,89,90,91,92,93,94,95,96,97,98,99,100,101,102,103,104,105,106,107,108,109,110,111,112,113,114]. Note: In the right figure, the order of the legend corresponds to the order of the pie chart sectors (starting clockwise from the 12 o’clock position).

Alkali activators are the fundamental component driving geopolymerization. Their composition and properties—such as modulus and concentration—significantly influence the type and performance of the resulting reaction products [12,35,117,122,123]. As shown in Figure 4, the activators in EGC are primarily liquid systems, with sodium hydroxide (NaOH) and sodium silicate (Na_2_SiO_3_) accounting for nearly 80%, making them the most widely used combination. Their combination effectively overcomes the limitations of single activators and enhances the overall reaction efficiency. NaOH provides a highly alkaline environment, rapidly breaking Si–O and Al–O bonds in the precursor materials and accelerating dissolution [124]. Meanwhile, Na_2_SiO_3_ supplements soluble silica and adjusts the Si/Al and Na/Al ratios by introducing Na^+^, further improving activation efficiency and promoting the formation of highly crosslinked gel networks [125,126].

**Figure 5:**
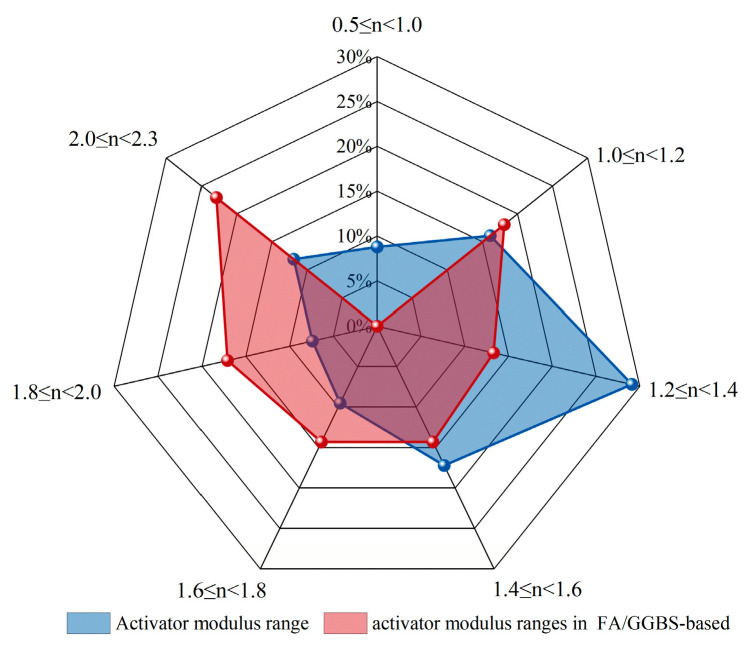
Statistical analysis of the distribution of activator modulus ranges in 285 valid EGC research cases and 105 valid FA/GGBS-based EGC research cases reported over the past five years [37,39,52,53,54,55,56,57,58,60,62,68,69,70,71,72,73,74,76,77,78,79,80,81,82,83,84,85,87,88,89,90,91,92,93,96,97,98,99,100,101,104,105,106,107,108,110,112,113].

**Figure 6:**
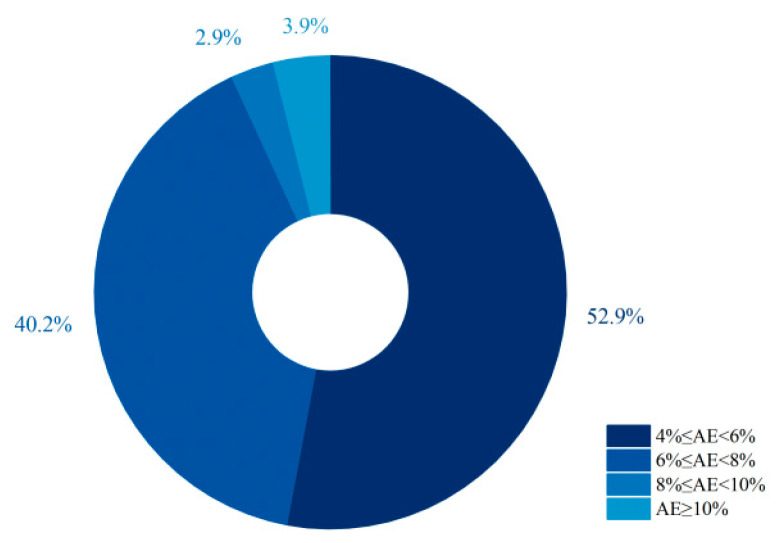
Statistical analysis of the proportion of alkali equivalent ranges in 102 valid EGC research cases reported in the past five years [37,52,55,62,70,72,78,79,85,92,96,98,99,100,105,106,107,108,110]. Note: The order of the legend corresponds to the order of the pie chart sectors (starting clockwise from the 12 o’clock position).

As shown in Figure 5, the modulus range is relatively wide, with successful studies reported across 0.5–2.3. Among them, the range of 1.2–1.4 is the most common, accounting for approximately 29.1% (nearly one-third) of the cases. As shown in Figure 6, the alkali equivalent is mostly concentrated between 4% and 8% [37,52,55,62,70,72,78,79,85,92,96,98,99,100,105,106,107,108,110], and activators within this range account for as high as 93.1%. For the most widely used fly ash–slag blended precursor systems, the modulus of the alkali activator is evenly distributed within the range of 1.0–2.3 (Figure 5) [39,52,53,54,55,60,71,77,78,81,83,84,87,91,92,98,104,107,108,112].

Apart from the mainstream alkali activators based on the combination of sodium hydroxide and sodium silicate, Figure 4 shows that anhydrous sodium metasilicate and sodium silicate solution have been used in many studies [64,65,70,72,75,79,99,101,110].

Aimed at practical engineering applications, research on powdered activators has gradually gained attention. These include anhydrous sodium metasilicate powder and sodium metasilicate pentahydrate, which can be used alone or in combination with sodium hydroxide granules. Under specific mix proportions, the performance of some powdered activators can match or even exceed that of liquid activators [53,56,80,88,95].

### 2.3. Sand

As an inert component, sand functions as the skeletal framework of EGC, bearing and transferring loads while restricting deformation of the binder matrix. However, its physical characteristics—particularly particle size—exert a significant influence on the uniaxial tensile performance of the composite [80,115,127,128].

According to studies by V. C. Li and others, the inclusion of sand affects the fracture toughness of the matrix. As particle size increases, fracture toughness tends to increase as well, due to mechanisms such as crack deflection and energy dissipation. While this may improve certain mechanical properties, it often leads to undesirable effects such as a reduced number of cracks and increased crack spacing, ultimately limiting the material’s tensile strain capacity. From the perspective of the energy criterion, a higher matrix toughness suppresses strain-hardening behavior, which is essential for EGC performance [8,115,129].

**Figure 7:**
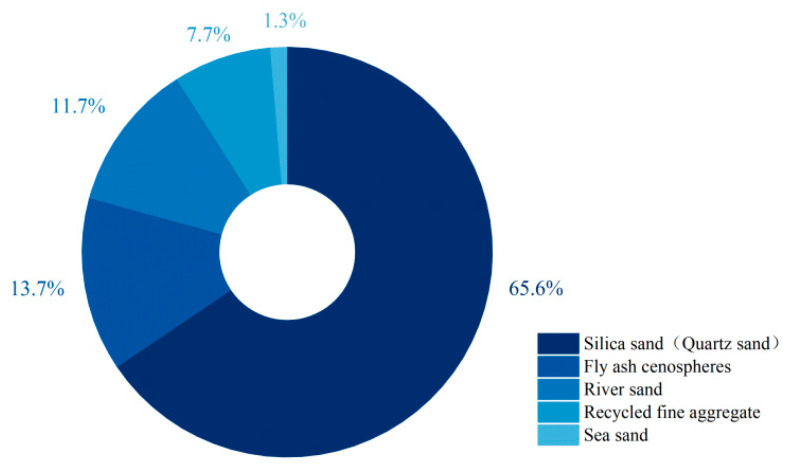
Statistical analysis of the proportion of fine aggregate types in 299 valid EGC research cases reported in the past five years [37,47,52,54,55,56,57,58,59,62,63,64,65,66,67,68,69,70,71,72,73,74,75,76,77,78,79,80,82,83,84,85,86,87,88,89,90,91,92,93,94,96,97,98,99,100,101,103,104,105,106,107,108,109,110,111,113,114]. Note: The order of the legend corresponds to the order of the pie chart sectors (starting clockwise from the 12 o’clock position).

Similar to ECC, EGC is a high-ductility material designed around microcrack control and thus is generally unsuitable for the incorporation of large aggregates. As shown in Figure 7, the most commonly used fine aggregate is silica sand (quartz sand) with a mean particle size of 100–300 μm [39,47,53,55,56,57,58,59,60,61,68,70,71,73,78,79,80,81,83,84,85,86,89,91,92,93,96,98,99,101,102,104,106,107,108,109]. This particle size strikes a balance—providing sufficient stiffness to suppress shrinkage while avoiding disruption of fiber dispersion. Additionally, due to the small sand-to-binder ratio, it prevents excessive increases in matrix toughness. However, the cost of silica sand is relatively high.

Many researchers have attempted to replace quartz sand with river sand and achieved favorable results, with the maximum particle size reaching 1000 μm [94,100,103,113,114]. In recent years, to reduce dependency on natural resources and promote circular utilization of waste, many researchers have investigated alternative aggregates, either partially or completely replacing silica sand. The references cited in this article [54,60,62,64,67,69,72,75,77,78,85,87,97,101,110,111] report a total of 54 successful cases of mixtures using alternative aggregates, covering industrial solid waste-based replacement aggregates such as fly ash cenospheres, steel slag, crushed brick sand, glass cullet sand, and recycled fine aggregates, as well as sea sand. These alternative aggregates have demonstrated favorable performance in both mechanical properties and environmental sustainability.

### 2.4. Fibers

#### 2.4.1. Type and Content of Fiber

The realization of strain-hardening in EGC depends not only on the matrix but more on the intrinsic mechanical properties and surface characteristics of the fibers. A wide variety of fibers have been used in fiber-reinforced composites, including steel fibers, basalt fibers, natural fibers, carbon-based fibers, and synthetic fibers.

Steel fibers are widely used due to their excellent mechanical properties, interface bonding, and thermal resistance. However, because of their high rigidity and strong interface bonding, steel fibers in EGC often lead to a reduced number of cracks and increased crack spacing, thereby increasing the risk of brittle failure and making it difficult to achieve the multi-crack strain-hardening behavior typical of EGC [88].

Basalt fibers are low-cost and thermally stable. Their inclusion can enhance the compressive and flexural strength of the material. When used alone, they can somewhat improve crack distribution, but their tensile strain capacity is generally below 1%, making it difficult to achieve the multi-crack strain-hardening behavior typical of EGC [130,131,132].

Natural fibers are also used in cementitious materials due to their environmental friendliness, renewability, and cost-effectiveness. However, the vast majority of natural fibers cannot achieve strain-hardening, and their tensile strain capacity is generally low [106,133,134].

Carbon fibers, due to their excellent mechanical, physical, and chemical stability, are representative high-performance reinforcing fibers and can be classified into microscale carbon fibers and carbon nanofibers. Compared with other fibers, carbon fibers are generally more expensive, and their high rigidity and strong interfacial bonding are unfavorable for achieving the typical multi-crack strain-hardening behavior in EGC. In contrast, carbon nanofibers (CNFs), particularly carbon nanotubes (CNTs) used in combination with PVA fibers, perform well in EGC. CNTs, formed by rolling single or multiple graphene sheets into tubular structures, possess a certain degree of flexibility, which helps improve crack distribution and enhance the material’s ductility [57,58,66,67,69,90,135].

Synthetic fibers, such as polyethylene (PE) and polyvinyl alcohol (PVA) fibers, are the most widely used reinforcing materials for achieving strain-hardening in engineered geopolymer composites (EGCs). These fibers are produced through polymerization and subsequent processing, exhibiting excellent strength, durability, and corrosion resistance. PE fibers possess high tensile strength and elastic modulus, are hydrophobic, and relatively cost-effective. In EGCs, they provide favorable pull-out behavior and energy dissipation, contributing to multi-crack formation and pronounced strain-hardening performance. PVA fibers, although more expensive, offer moderate strength and modulus, good ductility, and hydrophilic properties. However, their strong interfacial bonding may reduce crack numbers and increase crack spacing, thereby inhibiting strain-hardening. As a result, surface coating or hybridization strategies are often applied to modify their performance. Studies have shown that fiber hybridization or surface modification can significantly enhance EGC performance. For example, incorporating small amounts of steel fibers with low-modulus synthetic fibers can balance high strength and ductility [47,80,136]; blending PE with polypropylene (PP) can improve tensile strain capacity [90]; and surface modification of PVA or PE fibers with nano-SiO_2_ or nano-TiO_2_ can adjust fiber hydro-philicity/hydrophobicity and interfacial bonding, thereby optimizing crack distribution and enhancing strain-hardening behavior [55,108].

The most widely used and reliable fibers in EGC are PE and PVA fibers. By applying appropriate interfacial modifications to overcome their inherent limitations and then hybridizing the two, a synergistic effect can be achieved: PE provides strength and energy dissipation, while PVA contributes ductility and effective crack control, resulting in EGC with superior overall performance.

In ECC (and similarly in EGC), fiber content is not simply a case of “the more the better.” Instead, optimal performance results from careful coupling of fiber geometry (e.g., aspect ratio, surface texture) with matrix design (e.g., precursor type, aggregate size) and service conditions (e.g., load demands, durability requirements) [40,75,101].

**Figure 8:**
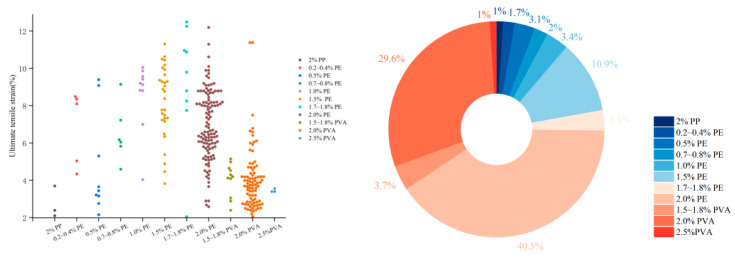
Statistical analysis of the relationship between fiber types, content and ultimate tensile strain in 294 valid EGC research cases reported in the past five years [37,39,47,52,53,54,55,58,60,61,62,63,64,65,68,70,71,72,73,74,75,76,77,78,79,81,82,83,84,85,86,87,88,89,91,92,93,94,95,96,97,98,99,100,101,102,103,104,105,106,107,108,109,110,111,112,113,114]. Note: In the right figure, the order of the legend corresponds to the order of the pie chart sectors (starting clockwise from the 12 o’clock position).

As shown in Figure 8, PE fiber, owing to their high elastic modulus, exhibits a relatively broad applicable content range. Through tailored matrix design and optimization, strain-hardening behavior can be achieved at fiber contents ranging from 0.2% to 2.0% by volume, with approximately 40.5% of studies adopting a 2.0% content. For PVA fibers, 1.8–2.0% is the most common range, due to its stronger interfacial bonding, which favors fiber rupture over pull-out, necessitating higher volume fractions to achieve sufficient crack-bridging capacity.

#### 2.4.2. Fiber Geometry

The geometry of fibers directly influences the interfacial bonding strength between fibers and the matrix, their dispersion, and orientation within the matrix, thereby significantly affecting the overall performance of EGC.

The cross-sectional shape and surface condition of fibers affect the contact area and frictional force at the fiber–matrix interface. Studies have shown that, to better achieve strain-hardening behavior, researchers have modified fiber geometry—fibers with rougher surfaces can enhance interfacial bonding through mechanical interlocking, while surface coatings may improve interfacial compatibility [55,137,138,139,140,141], thereby enabling higher strain-hardening capacity.

Fiber length is also critical. Longer fibers can form more effective bridges across cracks, but may lead to dispersion challenges and agglomeration. Conversely, shorter fibers are easier to disperse but less effective at bridging cracks [85,92,120,142,143,144]. Therefore, selecting an appropriate fiber length is essential for optimal EGC performance.

**Figure 9:**
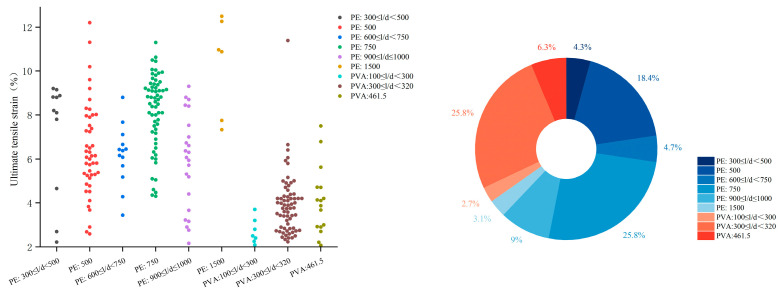
Statistical analysis of the relationship between fiber aspect ratios and ultimate tensile strain in 256 valid EGC research cases reported in the past five years [37,39,47,52,54,55,56,60,61,62,63,64,65,68,70,71,72,73,75,76,78,79,81,82,83,84,85,86,87,88,89,91,92,93,94,95,96,97,98,99,100,101,102,103,104,106,107,108,109,110,111,112,113,114]. Note: In the right figure, the order of the legend corresponds to the order of the pie chart sectors (starting clockwise from the 12 o’clock position).

The aspect ratio (length-to-diameter ratio) is another key parameter. A higher aspect ratio generally improves bonding due to increased surface area, but may impair dispersion and alignment during mixing. In most studies, PE and PVA fibers used in EGCs have lengths between 12–18 mm, with PE fiber diameter of 20–25 μm and PVA fiber diameter of around 40 μm [37,39,47,52,54,55,56,60,61,62,64,65,68,70,71,72,73,75,76,78,79,81,82,83,84,85,86,87,88,89,91,92,93,94,95,96,97,98,99,100,101,102,103,104,106,107,108,109,110,111,112,113,114]. As shown in Figure 9, about 48.9% of the studies used PE fibers with aspect ratios between 500–750, while PVA fibers predominantly had aspect ratios around 300.

## 3. Curing Regime

Curing regime is of critical importance for both traditional concrete and EGCs, as they directly determine the completeness and adequacy of hydration reactions and the quality of the hydration products, making curing a key factor in ensuring the overall performance of the material [52].

Unlike ECC, which uses Portland cement-based binders, EGC relies on geopolymer reactions, and often requires elevated temperatures—typically 50–80 °C—for optimal curing, especially for low-reactivity precursors like fly ash. High-temperature curing can significantly accelerate the breakdown of Si-O and Al-O bonds, facilitating the reorganization of [SiO4]^4−^ and [AlO4]^5−^ tetrahedral units. The experimental results indicate that the early strength under normal temperature curing may be only one-third to one-half of the strength under heat curing. If the temperature and humidity parameters are not suitable for the precursor system, rapid moisture evaporation can cause shrinkage cracks and may inhibit the formation of reaction products, which in turn leads to a reduction in compressive strength [145,146,147]. Considering the effect of defects, tensile ductility might actually increase [8,13]. Temperature also affects the interfacial interaction between fibers and the matrix. During the cooling process, the matrix undergoes thermal shrinkage, which can enhance the bonding with fibers [148].

Considering that traditional high-temperature curing consumes a large amount of energy, this runs counter to the original low-carbon concept of EGC materials and limits their practical application in engineering. Consequently, researchers have actively explored and adopted alternative curing regime. As shown in Table 1, segmented curing (e.g., initial heat treatment followed by normal temperature curing) and normal temperature curing are now the most commonly used approaches.

## 4. Mechanical Properties of EGC

### 4.1. Relationship Between Compressive Strength and Ultimate Tensile Strain of EGC

For any structural element, compressive strength remains a core performance indicator, with its importance reflected in aspects such as safety and durability. Similar to ECC, EGC is a material designed with the requirements of structural elements or components as its guiding principle, meaning that compressive strength remains the primary consideration. However, what distinguishes EGC from conventional concrete is its unique tensile behavior: the capacity for strain-hardening directly determines whether the material can meet the intended demands for ductility and crack control in structural elements or components. Therefore, tensile strain capacity is also a critical parameter for evaluating EGC performance.

Compressive strength and the tensile stress–strain relationship are fundamental characteristics of EGC materials, making it highly valuable to investigate the relationship between them. This review compiled studies from the past five years in which uniaxial ultimate tensile strain exceeded 2% and plotted the relationship between compressive strength and strain-hardening behavior.

**Figure 10:**
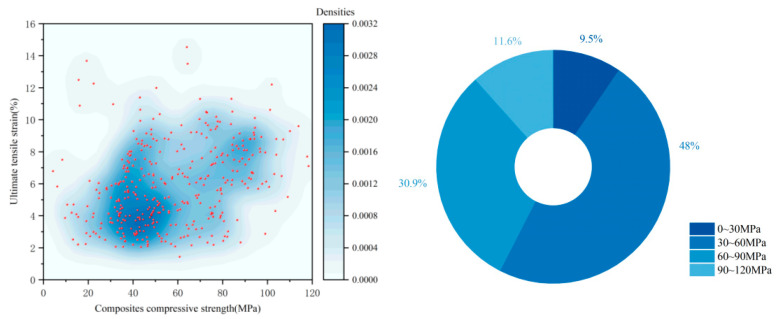
Statistical Analysis of the Compressive Strength–Ultimate Tensile Strain Relationship in 379 Valid EGC research cases Reported in the Past Five Years [37,39,47,52,53,54,55,56,57,58,59,60,61,62,63,64,65,66,67,68,69,70,71,72,73,74,75,76,77,78,79,80,81,82,83,84,85,86,87,88,89,90,91,92,93,94,95,96,97,98,99,100,101,102,103,104,105,106,107,108,109,110,111,112,113,114,119]. Note: In the right figure, the order of the legend corresponds to the order of the pie chart sectors (starting clockwise from the 12 o’clock position).

As shown in Figure 10, the statistics show that approximately half of the EGCs have compressive strengths in the range of 30–60 MPa (It can account for 48% of the statistical sample.), with an overall range spanning 20–120 MPa. These strength levels not only meet the requirements of most engineering applications, such as prefabricated structural components (non-prestressed may require ≥40 MPa; prestressed may require ≥60 MPa), repair mortars (generally needs to be ≥30 MPa) and seismic joints (generally needs to be ≥20 MPa), but also maintain good ductility, making them suitable for a wide range of specialized structural demands.

### 4.2. Relationship Between Matrix Compressive Strength and Ultimate Tensile Strain of EGC

This study also investigated the matrix strength of EGC. Due to the limited number of reports on matrix strength, data from the past decade were additionally collected. The results show that approximately 64.7% of matrix strengths fall within the range of 25–50 MPa. An initial estimation of matrix strength can serve as a useful design indicator to increase the likelihood of achieving strain-hardening.

The type of precursor plays a decisive role in the design and performance regulation of EGC, as its composition, reactivity, and particle morphology directly influence the reaction process and the types of gel phases formed, thereby significantly affecting the mechanical properties and interfacial compatibility of the material [24]. Different precursor types produce distinct primary gel phases; for example, fly ash predominantly forms N-A-S-H gels, while blends with slag result in interwoven N-A-S-H and C-A-S-H gels. Moreover, the precursor type affects the synergistic effect of fiber reinforcement in EGC. For instance, under PVA fiber reinforcement, fly ash–slag blended systems exhibit greater improvements in tensile strain capacity compared to pure slag systems [53].

**Figure 11:**
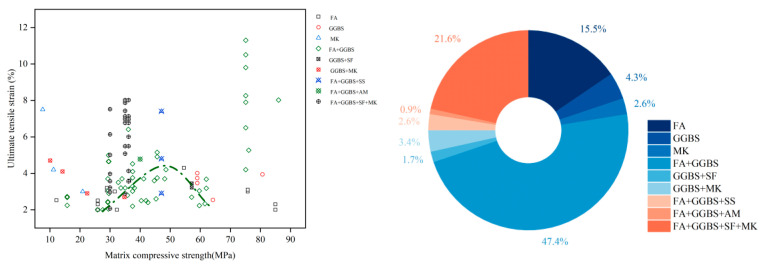
Statistical analysis of the matrix compressive strength–ultimate tensile strain relationship in 116 valid EGC research cases reported in the past ten years [47,51,55,57,58,62,63,67,68,71,76,82,87,88,92,93,104,106,107,108,112,115,116,117,118,119,120,121]. Note: In the right figure, the order of the legend corresponds to the order of the pie chart sectors (starting clockwise from the 12 o’clock position).

**Figure 12:**
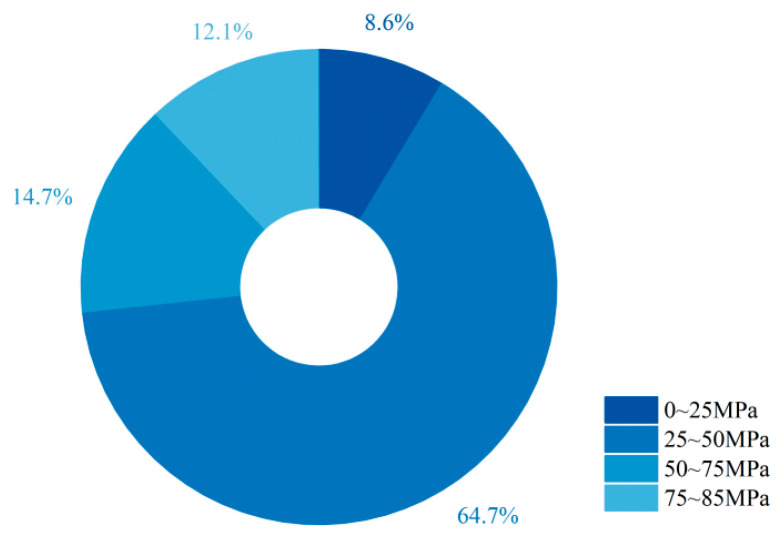
Statistical analysis of the proportion of matrix compressive strength ranges in 116 valid EGC research cases reported in the past ten years [47,51,55,57,58,62,63,67,68,71,76,82,87,88,92,93,104,106,107,108,112,115,116,117,118,119,120,121]. Note: The order of the legend corresponds to the order of the pie chart sectors (starting clockwise from the 12 o’clock position).

Based on the results reported in the literature, we classified EGCs according to the precursor combinations in the matrix, as shown in Figure 11 and Figure 12. For most fly ash–slag blended systems, strain-hardening with ultimate tensile strain ≥2% corresponds to matrix strengths in the range of 30–50 MPa. In fly ash–slag blended systems with matrix strengths between 25 and 60 MPa, a general trend is observed: strain-hardening capacity initially increases with matrix strength and then gradually decreases. A similar trend is also seen in systems where fly ash is used as the sole precursor.

## 5. Conclusions

The EGC systems reported over the past five to ten years with uniaxial ultimate tensile strains ≥ 2% statistically analyzed, focusing on material composition, curing regime, and the relationship between (matrix) compressive strength and tensile performance. The following conclusions can be drawn:

(1) Strain-hardening behavior in EGC is achieved by tuning precursor type, activator composition, fiber characteristics, and interfacial properties. Generally, a low-toughness matrix, fibers with moderate modulus and strength, and weak interfacial bonding are conducive to achieving strain-hardening and multiple cracking.
(2) The most widely used precursor system is the fly ash–slag blend, activated by a combination of sodium hydroxide and sodium silicate. Effective systems typically use activators with a modulus of 1.4–1.8 and alkali equivalent of 4–8%. Common fibers include PE (2.0%, aspect ratio 500–750) and PVA (1.8–2.0%, aspect ratio ≈ 300). Fine aggregates are primarily silica sand with particle sizes of 100–250 μm. Curing strategies now favor normal temperature or segmented curing, depending on precursor type.
(3) The compressive strength of EGC ranges from 20–120 MPa, satisfying structural requirements. Most high-performance EGCs achieving ultimate tensile strains ≥ 2% have matrix strengths between 25–50 MPa. For FA-GGBS and fly ash-only systems, tensile performance tends to improve with increasing matrix strength, peaking at an optimal range before declining.

Future Research Prospects: Although EGC has made significant progress in performance optimization and greening, it is still complex and influenced by many factors, facing numerous challenges. Future research should focus on the following directions:

(1) Green Design: Promote research on precursors and powder activators based on solid waste resources, explore the synergistic mechanisms among various solid wastes, and develop a low-carbon, sprayable, and pumpable engineering-applicable EGC mix system.
(2) Engineering Applications: Conduct service performance evaluations under the coupling of multiple environmental factors and verify applications at the structural component level, achieving the transition of EGC from materials research to engineering practice.

Through the synergistic optimization of material composition, structure, and performance, EGC is expected to show broad application prospects in future green buildings and the enhancement of infrastructure durability.

## Figures and Tables

**Table 1:** Statistical analysis of different curing regimes in 52 EGC research cases reported in the past five years.

Presursor and Activator of Matrix of 52 EGC Cases	Curing Regime
FA (SH, SMP) [61,95]	High temperature curing + normal temperature curing (cure 5 d at 23 ± 3 °C demolding, 36/48 h at 80 °C, 23 ± 3 °C to 28 d).
FA (SH, SSI/SMA, SSI) [79,89,93,112]	High temperature curing + normal temperature curing (60 °C 8 h/70 °C 24 h/90 °C 72 h, room temperature to 28 d); Normal Temperature Curing (room temperature to 7 d).
GGBS, SF (SH, SSI) [62,94,103,113]	Normal temperature curing (room temperature/20 °C, 95% R.H. to 28 d); High temperature curing + normal temperature curing (60 °C 1 d, 20 °C, 60% R.H. to 28 d; 80 °C 2 h, Air-cured to test); Low temperature curing (−5 °C to 28 d).
FA, GGBS (SMA) [53,56,80]	Normal temperature curing (room temperature/20 ± 2 °C, 95% R.H. /25 °C, ≥80% R.H. to test).
FA, GGBS (SH, SSI) [39,47,52,54,55,60,68,71,77,78,81,84,86,87,91,92,98,104,107,108,109,111,112,114]	Normal temperature curing (standard curing room (20 ± 2 °C, 95% R.H.)/curing room (20 ± 3 °C, ≥90% R.H.)/23 ± 2 °C, 95 ± 5% R.H./room temperature to test); High temperature curing + normal temperature curing (60 °C 24 h, standard curing to test; 70 °C 24 h demolding, room temperature to 28 d; 24 h demolding, 80 °C to 3 d; plastic wrap sealing, 80 °C 2 h, room temperature to test; constant temperature (20 °C) and humidity 24 h demolding, 60 °C/80 °C constant temperature and humidity to test); Curing in water (curing in water to test).
FA, GGBS, SF (SMA + SSI) [70,72,99]	High temperature curing + normal temperature curing (80 °C 72 h remove to test); Normal temperature curing (room temperature to 28 d).
FA, GGBS, SF (SH + SSI) [59,74,85,86]	High temperature curing + curing in water (48 h demolding, 100 °C 24 h, 23 ± 3 °C curing in water to 28 d); Normal temperature curing (room temperature (20 ± 2 °C, 95% R.H.) to test).
FA, GGBS, SSL (SH + SSI) [37,73,111]	Curing in water (curing in water to test); Normal temperature curing (20~25 °C to test).
MK, SF/MK, RCP/MK, SF, GGBS (SMA + SSI) [47,64,110]	High temperature curing + normal temperature curing (80 °C 24 h, room temperature to 3/7 d).
FA, GGBS, SF, MK (SH + SSI) [57,66,67,69,90]	Normal temperature curing (standard curing room/box (20 ± 2 °C, ≥95%R.H.); Standard curing room (17~23 °C, ≥90% R.H.) to 28 d).

Note: FA: fly ash; GGBS: ground granulated blast furnace slag; SF: silica fume; SSL: steel slag; MK: metakaolin; RCP: Recycled concrete powder; SH: Sodium hydroxide; SSI: sodium silicate; SMP: sodium metasilicate pentahydrate; SMA: sodium metasilicate anhydrous.

## Data Availability

No new data were created or analyzed in this study. Data sharing is not applicable to this article.

## Abbreviations

The following abbreviations are used in this manuscript:

EGC	Engineered geopolymer composites
ECC	Engineered cementitious composites
FA	Fly ash
GGBS	Ground granulated blast furnace slag
RBWP	Recycled brick waste powder
SF	Silica fume
MK	Metakaolin
RHA	Rice husk ash
RCP	Recycled concrete powder
RPP	Recycled paste powder
RBP	Recycled brick powder
SSL	Steel slag
RM	Red mud
AM	Alkaline mud
SAP	Superabsorbent polymers
SSI	Sodium silicate
SH	Sodium hydroxide
SMA	Sodium metasilicate anhydrous
SMP	Sodium metasilicate pentahydrate
CH	Calcium hydroxide
SSU	Sodium sulfate
